# Possible association of *fimA* genotype of *Porphyromonas gulae* with the severity of periodontal disease and the number of permanent teeth in dogs

**DOI:** 10.3389/fvets.2023.1022838

**Published:** 2023-02-06

**Authors:** So Shirahata, Naoki Iwashita, Rie Sasaki, Ryota Nomura, Masaru Murakami, Junya Yasuda, Hidemi Yasuda, Kuniyasu Nakajima, Hiroaki Inaba, Michiyo Matsumoto-Nakano, Kazuhiko Nakano, Jumpei Uchiyama, Tomoki Fukuyama

**Affiliations:** ^1^Laboratory of Veterinary Pharmacology, School of Veterinary Medicine, Azabu University, Sagamihara, Kanagawa, Japan; ^2^Primo Animal Hospital Sagamiharachuo, Sagamihara, Kanagawa, Japan; ^3^Bioalchemis, Fuchu, Tokyo, Japan; ^4^Department of Pediatric Dentistry, Osaka University Graduate School of Dentistry, Suita, Osaka, Japan; ^5^Laboratory of Molecular Biology, School of Veterinary Medicine, Azabu University, Sagamihara, Kanagawa, Japan; ^6^SPECTRUM LAB. JAPAN Co., LTD., Yasuda Veterinary Clinic, Tokyo, Japan; ^7^AlphaVets Co., LTD., Yasuda Veterinary Clinic, Tokyo, Japan; ^8^Nakajima Animal Hospital, Tokyo, Japan; ^9^Department of Pediatric Dentistry, Okayama University Graduate School of Medicine, Dentistry and Pharmaceutical Sciences, Okayama, Japan; ^10^Department of Bacteriology, Graduate School of Medicine Dentistry and Pharmaceutical Sciences, Okayama University, Okayama, Okayama, Japan

**Keywords:** dog, periodontal disease, *Porphyromonas gulae*, *fimA* type, number of permanent teeth

## Abstract

Previous research has demonstrated that *Porphyromonas gulae (P. gulae)* significantly contributes to the development of periodontal disease in dogs. *Porphyromonas gulae* is divided into three subtypes according to the 41-kDa filamentous appendage (*fimA*), defined as types A, B, and C. This study aimed to elucidate the association between *fimA* type of *P. gulae* with the number of permanent teeth, reflecting the severity of periodontal disease. Two hundred twenty-five dogs were categorized by *P. gulae fimA* type as negative, type A dominant, type B dominant, and type C dominant. The stage of periodontal disease in *P. gulae*-positive dogs increased with age, particularly in type C dominant dogs. Correspondingly, the number of permanent teeth in *P. gulae fimA* type C-dominant dogs was significantly lower than that of *P. gulae*-negative dogs, suggesting there is a significant association between *fimA* type of *P. gulae* and the number of permanent teeth resulting from the development of periodontal disease.

## Introduction

Periodontal disease is extremely common in dogs and is associated with serious systemic diseases (1–6). Studies have reported that 84% of Beagles over the age of 3 and 100% of Poodles over the age of 4 develop periodontal disease (5–9). Current living environment, type and frequency of dental care, and infectious diseases such as viral and bacterial diseases are involved in the progression of periodontal disease. However, the mechanisms underlying the development of periodontal disease in dogs require further investigation.

Recently, *Porphyromonas gulae* (*P. gulae*), a gram-negative black pigmented anaerobic bacterium, was identified as one of the possible pathogens that causes periodontal disease in dogs, and it causes the onset and exacerbation of periodontitis (10). *Porphyromonas gulae* can be subtyped into three groups according to the genotype of Fimbrillin (*fimA*; a pathogenic protein that forms fimbria on the surface of the bacterium): types A, B, and C (11, 12). *FimA* is expressed on the cell surface of *P. gulae* and plays a pivotal role in adherence to the teeth and may induce the pathogenic features of periodontitis (11, 12). Studies in a mouse abscess model demonstrated that type B causes significantly greater systemic inflammation than type A. Moreover, type C induces even higher systemic inflammation than the other genotypes (11, 12). Indeed, type C is considered to be the most virulent of the genotypes, as it is predominant in the oral cavities of dogs with severe periodontitis (12, 13). However, previous studies only demonstrated *fimA* Type C may be found more frequently in severe disease samples and have the ability to cause more inflammation than other types in a mouse model, this does not prove causation of periodontitis in dogs. Therefore, the association between *fimA* type and clinical features of periodontal disease is not yet fully understood. The present study aimed to elucidate the possible association between *fimA* types of *P. gulae* and the number of permanent teeth resulting from the severity of periodontal disease in dogs. We hypothesized that clinical features of canine periodontitis and the number of permanent teeth were linked with *fimA* types of *P. gulae*, as seen in the murine study described above, and that type B and C were more virulent than type A.

## Methods

This study was performed cross-sectionally, and all protocols of this study were conducted in accordance with the Animal Care and Use Program of Azabu University (Approval No. 200318-1). The study was explained to all owners, who provided written informed consent for approval of their pets participation. Severity of periodontal disease and number of permanent teeth were compared between genotypes of *fimA* in each age group. Oral swab specimens were collected from the gingival margin of the maxillary right or left canine and fourth premolar using a microbrush (Microapplicator fine, FEED Corporation, Yokohama, Japan) as described previously (10). Detection of *P. gulae* and genotype of *fimA* were determined using previously described PCR-based methods (11, 12). Periodontal disease stage was grossly evaluated according to previously described criteria (American Veterinary Dental College. https://avdc.org/avdc-nomenclature/) under general anesthesia and categorized as follows: normal (clinically normal), Stage 1 (gingivitis only, without attachment loss), Stage 2 (early periodontitis), Stage 3 (moderate periodontitis), and Stage 4 (advanced periodontitis). Disease stage was determined based on the most severely affected tooth (typically the maxillary premolar). Clinicians participating in this study had longer than 10 years clinical experience and were trained and certified by the special course of Small animal dental society of Japan. For the periodontal disease stages and number of teeth, data are expressed as the mean ± standard error of mean. After the normality of dependent variables and homogeneity of variances have been confirmed by Shapiro-Wilk test and Brown-Forsythe test, respectively. The relation of periodontal disease stages and presence/absence of *P. gulae* were analyzed as a two-way analyses of variance (ANOVA) design (Type III sum-of-squares), followed by Šídák's multiple comparisons test. Secondary, the relation of periodontal disease stages and various genotype groups were analyzed as a two-way ANOVA design (Type III sum-of-squares), followed by Dunnett's multiple comparison tests (comparison with negative). For number of residual teeth, as the complete lack of variation in the <50 months *P. gulae*-negative group, to dichotomize the dependent variable as full dentition vs. <42 teeth and run the analysis as a logistic regression in only 50–100 months and >100 months age groups. Statistical significance was estimated at 5 and 1% levels of probability (Prism 9, GraphPad Software, San Diego, CA). Power analysis has also been performed to confirm the sample size of this study was appropriate. The proportion of various genotype groups in each age group were analyzed with a Chi square test. There was no significant interaction between factors in every ANOVAs.

## Results

**Figure 1:**
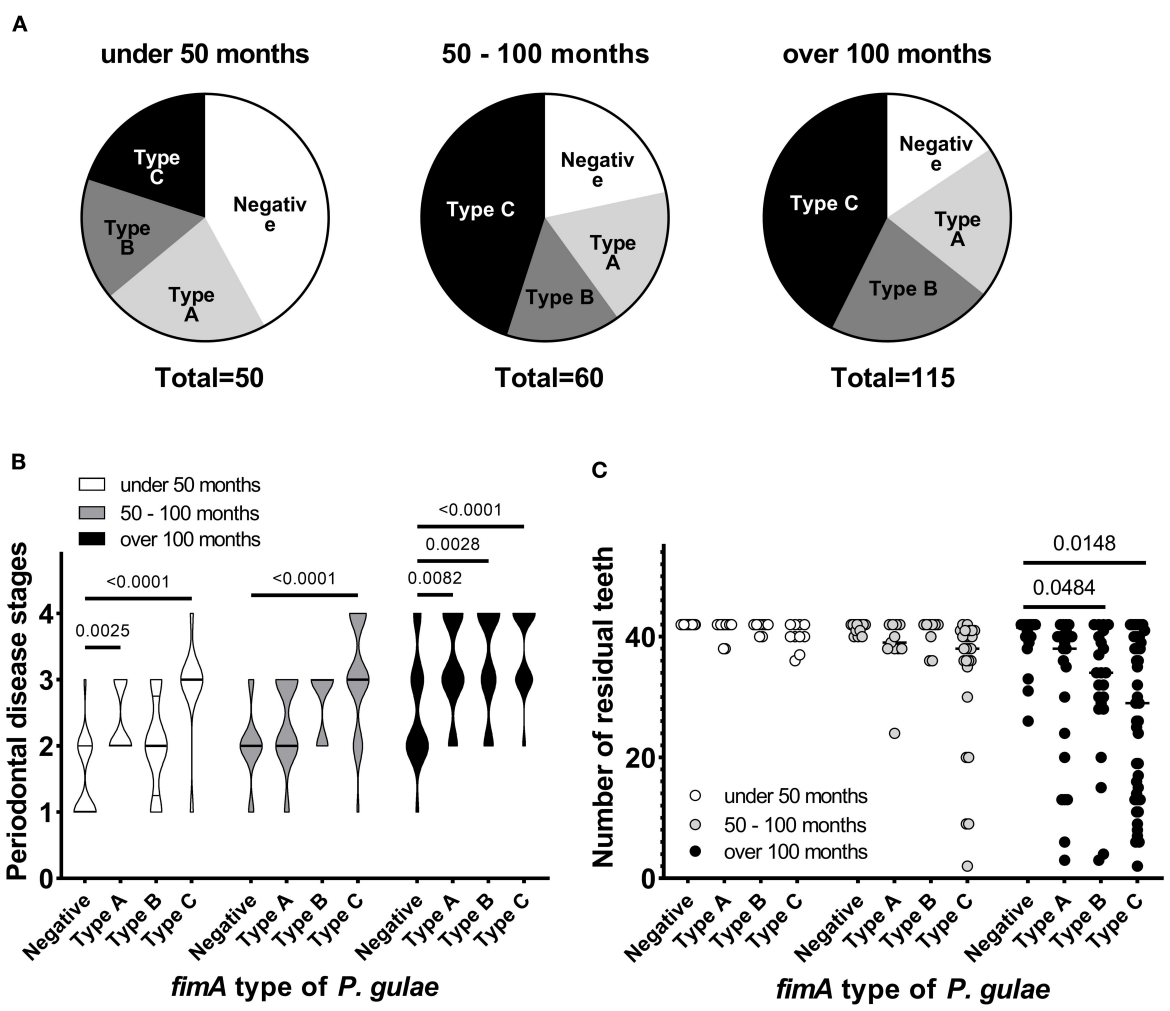
Contribution of *fimA* type of *Porphyromonas gulae* to periodontal disease. **(A)** *Porphyromonas gulae* carriage and associated genotype. **(B)** Association between periodontal disease stage and *fimA* type of *P. gulae* in each age group (Dunnett's multiple comparison test vs. the *P. gulae*-negative group). **(C)** Association between *fimA* type of *P. gulae* and the number of permanent teeth in each age group (a logistic regression analysis between full dentition and <42 teeth in 50–100 and >100 months age groups).

**Figure 2:**
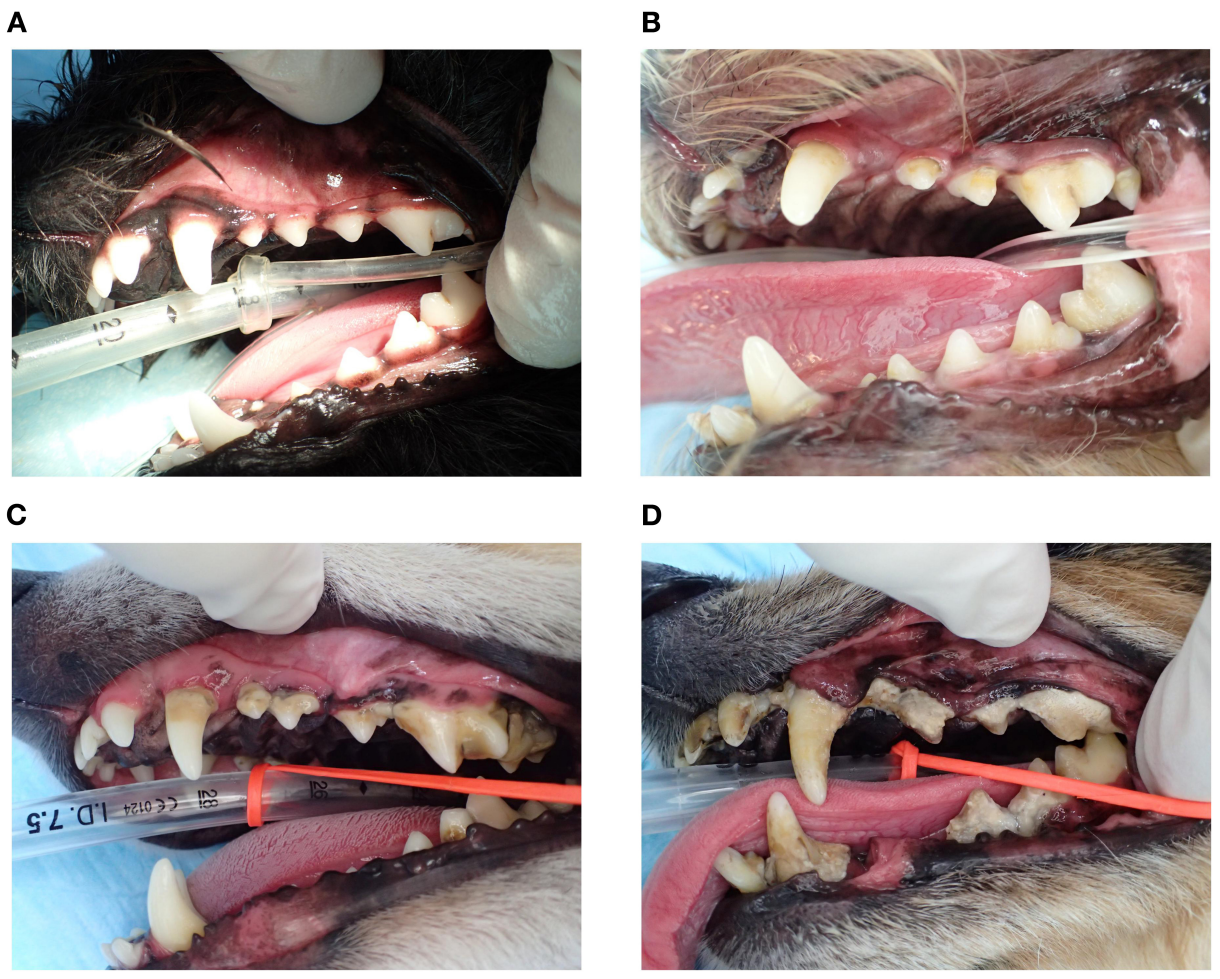
Representative image of gingival and teeth of dogs in the 50–100-months age group. **(A)** A subject negative for *Porphyromonas gulae*, **(B)** *fimA* -type A *P. gulae*, **(C)** *fimA* type B *P. gulae*, and **(D)** *fimA* type C *P. gulae*.

The study was performed among 225 dogs (age 6–211 months, dog breed: toy poodle, chihuahua, dachshund, maltese, pomeranian, yorkshire terrier, miniature schnauzer, papillon, and mix) with mild-to-severe periodontal disease, at five Japanese animal clinics (Primo Animal Hospital Sagamiharachuo, Sagamiono Primo Animal Hospital, Atsugi Primo Animal Hospital, Yasuda Veterinary Clinic, and Nakajima Animal Hospital). Dogs with a recent (last 6 months) history of antibiotics usage were excluded from the study. Dogs were divided into three groups based on age: <50 months (~4 years; *n* = 50), 50–100 months (~4–8 years; *n* = 60), and >100 months (~8 years; *n* = 115). The proportion of *P. gulae*-positive dogs increased with age (Figure 1A). In particular, the proportion of *P. gulae fimA* type C-dominant dogs significantly increased with age compared to other genotypes (20% in <50 months group, >40% in 50–100 and >100 months groups). The proportion of *P. gulae fimA* type C-dominant dogs is higher in the 50–100 and >100 month groups vs. the <50 month group. When we compared the severity of periodontal disease between presence and absence of *P. gulae*, a significant increase was observed in dog with *P. gulae* compared to *P gulae*-negative dogs in all age groups. When we compared the severity of periodontal disease between genotypes (Figure 1B), a significant (compared to the negative group) increase was observed in dogs with dominant *fimA* type A and *fimA* type C compared to *P. gulae*-negative dogs in the <50-months age group (the PD stage of type C was twice as many as that of the negative group). In the 50–100-months age group, significant increases (~50% of the PD stage was increased compared to the negative group) were observed in *fimA* type C. In the >100-months group, the presence of all genotypes of *P. gulae* significantly increased the stage of periodontal disease compared to that in *P. gulae-*negative dogs. Strikingly, *P. gulae* type C infections significantly associated with periodontal disease stages at all ages directly or indirectly (there is a possibility other bacterium in the community could be being influenced by *P. gulae*). Finally, the association between the genotype of *P. gulae* and the number of permanent teeth was examined (Figure 1C). According to the results of a logistic regression analysis between full dentition and <42 teeth in 50–100 and >100 months age groups, the number of dogs with full dentition in *P. gulae*-positive groups decreased with age, however, a significant decrease was observed only in dogs with dominant *fimA* type B and C compared to *P. gulae*-negative dogs in the >100-months group (Figure 2 shows representative image of dogs in the 50–100 months). These findings indicate that there is a significant association between the presence of *P. gulae* and the number of permanent teeth.

## Discussion

To the best of our knowledge, this is the first epidemiological study to demonstrate a link between the genotype of *P. gulae* and the number of permanent teeth as a clinical feature of periodontal disease. In the early life stage (under 50 months), there are significant impacts of *fimA* type C infection on periodontal disease stages compared to the negative group. Adverse impact of *fimA* type C was also demonstrated according to the significant increase of periodontal disease stage and the significant decrease in permanent teeth in older stages (50–100 months and over 100 months) within this genotype. On the other hand, it is still uncertain why *fimA* type C is increasing with advancing age since there is no longitudinal knowledge to know if *fimA* Type changes within an individual over time. The proportion of other types of *fimA* (A and B) is not influenced by age, therefore, it is unlikely *fimA* type A and B are changed to type C during life. Possible transition of *fimA* type within an individual over time is under investigation as a next step of this project in our group.

As an initial step, *P. gulae fimA* genotype should be diagnosed as early as possible, as this information could alert pet owners and veterinarians to take all necessary steps to control the development of the disease. Our findings clearly indicate that *P. gulae* genotype is associated with periodontal disease severity. Thus, the genotyping of *P. gulae fimA* type during basic check-ups might be a future option better to understand the oral environment. In all advanced cases of periodontal disease, appropriate dental care should be undertaken to avoid further disease development. It has been reported that *P. gulae* is susceptible to clindamycin, which has been approved for the treatment of periodontal disease in dogs (14, 15). Recently, our group showed that combination therapy using clindamycin and IFN-α treatment can improve periodontal condition and reduce *P. gulae* in dogs (13).

Taken together, our findings demonstrate that there is significant association between the presence of *P. gulae fimA* type C and the reduced number of permanent teeth resulting from the development of periodontal disease. However, it should be noted that there are several limitations in the current report including reliance on the score for the most severe tooth in the mouth to reflect periodontal disease stage, and no longitudinal knowledge to know if *fimA* Type changes within an individual over time.

## Data availability statement

The datasets presented in this study can be found in online repositories. The names of the repository/repositories and accession number(s) can be found in the article/ supplementary material.

## Ethics statement

The animal study was reviewed and approved by Animal Care and Use Program of Azabu University. Written informed consent was obtained from the owners for the participation of their animals in this study.

## Author contributions

SS, NI, RN, MM, JY, HY, HI, MM-N, KN, JU, and TF contributed to the study conception and design. SS, NI, RS, RN, MM, JY, HY, HI, KN, MM-N, KN, JU, and TF performed material preparation, data collection, and analysis. TF wrote the first draft of the manuscript. All authors commented on the earlier versions of the manuscript, read, and approved the final manuscript.
